# Analysis of the influence of educational level on the nutritional status and lifestyle habits of the young Spanish population

**DOI:** 10.3389/fpubh.2024.1341420

**Published:** 2024-04-08

**Authors:** Elena Sandri, Juan Pardo, Eva Cantín Larumbe, Germán Cerdá Olmedo, Antonio Falcó

**Affiliations:** ^1^Faculty of Medicine and Health Sciences, Catholic University of Valencia San Vicente Mártir, Valencia, Spain; ^2^Doctoral School, Catholic University of Valencia San Vicente Mártir, Valencia, Spain; ^3^Embedded Systems and Artificial Intelligence Group, Universidad Cardenal Herrera-CEU, CEU Universities, Valencia, Spain; ^4^Degree in Data Science, Polytechnical University of Valencia, Valencia, Spain; ^5^Department of Mathematics, Physics and Technological Sciences, Universidad Cardenal Herrera-CEU, CEU Universities, Valencia, Spain

**Keywords:** nutrition assessment, nutrition surveys, habits, young adult, educational status, public health, clustering analysis

## Abstract

**Aim:**

This study aims to analyze some nutrition and health habits of young people and the impact of educational attainment on health.

**Methods:**

An observational, descriptive, and cross-sectional study was carried out using surveys. Using non-probabilistic snowball sampling, a previously validated questionnaire was disseminated through networks, collecting a sample of 9,681 people between 18 and 30  years old. Comparative analyses between groups were obtained by clustering and the corresponding statistical tests.

**Results:**

The results showed how young people with higher education generally have a lower BMI, a higher healthy nutrition index, less frequent consumption of sugary drinks, and less smoking than their peers with basic education. These healthier habits are reflected in the higher self-perceived health status of the higher-educated group. While for all the educational levels analyzed, the minutes of physical activity practice are above the 150 min recommended by the WHO.

**Conclusion:**

Our findings suggest that young people’s education level is of fundamental importance for health, particularly for nutritional habits. In general, the lifestyle habits of the young Spanish population are healthy, but there is a need for improvement in those aspects related to nutrition and food.

## 1 Introduction

Habits can be defined as the frequent repetition of meaningful action patterns in a stable environment (1). They can be learned through “habit learning” or can also be shared and institutionalized in the form of rituals (2). Habits can change due to alterations in our environment, which can lead to the formation of new habits. Psychosocial factors such as self-efficacy, intrinsic motivation, social support, and extrinsic motivation may also influence the formation of new habits (3).

At the age at which the individual grows and forms, childhood, and adolescence are considered fundamental stages of maturing (4). However, there is another period in a person’s life of great importance in the development of routines and behavioral habits that will later affect the health and well-being of the individual, and that is the period of youth. This period coincides with significant changes: leaving the family home to live more independently (5), a change of schedule that in many cases leads to a reduction in physical activity (6) or the creation of new friendships and, with it, often new fun routines (7).

In 1985, the United Nations General Assembly set the age range of youth to be 15 to 24 years. In the last 35 years, society has changed considerably. The quality of life in industrialized countries has increased, as has life expectancy, and these limits have lengthened (8). In fact, according to the 2016 Report on Youth in Spain (9), a young person is considered to be a young person up to 30, while the Spanish National Institute of Statistics sets this limit between the ages of 15 and 29 inclusive.

The habits that are acquired during youth can have a significant impact on adult behavior. Habits can become automatic and routine and can be influenced by personality traits such as impulsivity and compulsivity (1). A study found that social media habits among youth can impact their social capital (10). Health-related lifestyles acquired during youth can affect a person’s health in the long term (11). Furthermore, sedentary behavior among children and youth can lead to prolonged sitting hours and excessive screen-based sedentary time, which can carry into adulthood (12). Habits can also be taught through example and training, and educators play a crucial role in this field.

Eating and nutritional habits are among the most important due to their direct impact on health. An incorrect diet can lead to the appearance of a whole series of diseases, even serious ones, such as obesity (13) and cardiovascular diseases (14), among others.

A recent systematic review of young people’s dietary habits found very varied results, pointing to the complexity and difficulty of studying the dietary habits of a population (15). The analyzed articles found that young people generally show poor adherence to the Mediterranean diet, consume fruit and vegetables below the dietary recommendations, and ingest unhealthy foods such as fried, ultra-processed, or fast food more frequently than recommended (16–19). The review also pointed to a vast difference between studies regarding other habits such as the number of meals per day, frequency, and type of breakfast or eating with the family (20–22).

Another review (23), which also studied adolescents’ nutritional and sustainability habits, focused more on analyzing the type of instrument used to record and study these habits. The authors found the use of well over 64 instruments to measure dietary habits, 14 different ones centered only on measuring adherence to the Mediterranean diet. This wide variety of instruments and methods is perhaps why finding a common and agreed explanation for the variability of results obtained on nutrition and health habits even within the same country is still challenging.

Given the importance of acquiring balanced and healthy nutrition and lifestyle habits in youth and maintaining them throughout life, it is crucial to investigate the factors that may influence these habits to intervene where possible.

One factor that seems to have a significant influence is the level of education and training, suggesting that people with more education may be more critical of advertising and food industry trends (24, 25) or better able to understand and evaluate nutritional information on food labels (26, 27). This knowledge might help them to make healthier choices when purchasing and consuming certain foods or products.

A correct choice of food is perhaps the differential element for adequate nutrition in a country like Spain, where both the availability and affordability of food and its general safety are already good. The Global Food Security Index 2022 (28), which analyses food security around the world, highlighting future challenges for improvement and developing solutions, has placed Spain in the 20th position out of the 113 countries studied.

Moreover, people with higher education generally have greater access to educational resources, such as books, webinars, videos or podcasts on nutrition and health, which leads to greater knowledge and more reliable information in this field. Increased awareness of the importance of long-term health can motivate them to adopt healthier habits and make informed dietary and lifestyle choices based on evidence and scientific knowledge (29, 30).

Augmented education and knowledge in nutrition and health can help to successfully address what the FAO, in its latest report on the state of food security and nutrition in the world in 2023 (31), defines as one of the most significant global challenges in nutrition. Ensuring that the increasing availability of 4 and 5th-range foods, primarily rich in fats, preservatives, additives, and other unhealthy substances, does not automatically lead to the assumption by consumers of a high-calorie diet that is poor in nutrients and vitamins and detrimental to their health.

The Spanish education system is currently regulated by the Organic Law 3/2000, which replaced the previous Organic Law on Education (LOMLOE) (32) and is divided into different educational stages.

It starts with primary education (between 6 and 12 years old), which is compulsory, and continues with secondary education, which is also compulsory (ESO, *Educación Secundaria Obligatoria*), and consists of 4 years (between 12 and 16 years old). After the ESO stage, different educational stages begin, which are already optional and which students can choose if they wish. *Bachillerato*, or alternatively professional training (2 or 3 years), is the access route to a University Degree (usually 4 years) which can be complemented with a master’s degree. Finally, the highest university degree is the PhD, which generally focuses on pursuing a professional career in the academic world.

Therefore, this research aims to explore the influence of the young Spanish population’s education level on their nutrition and lifestyle habits. The study of this relationship will allow us to discover patterns and highlight possible specific areas for action in the field of public health to improve those habits or aspects that are found to be less healthy.

## 2 Materials and methods

### 2.1 Type of study and sampling

A cross-sectional study was conducted on the young Spanish population (18–30 years old) residing in Spain, excluding those persons with chronic diseases or temporary situations that could affect their diet.

### 2.2 Instrument

A self-developed questionnaire was developed and validated with the help of a pilot group of 52 persons and a nominal group of seven experts in the field of health. The expert group, composed of two psychologists, a nutritionist, a social educator, two family doctors, and a communication professional, approved the instrument’s final version after reviewing the questionnaire and the pilot group’s results.

The questionnaire, provided using Google Forms, was disseminated through social networks using a non-probabilistic snowball sampling. The main dissemination channel was Instagram, where the account “@elretonutricional” was created, from which various professionals, influencers, and people who supported the dissemination were contacted. The researchers’ networks were also used (LinkedIn, Twitter, WhatsApp, and Facebook), and emails were sent to different establishments throughout Spain, specifically selected for their heterogeneous public (pharmacies, tobacconists).

### 2.3 Variables

The questionnaire collects sociodemographic and anthropometric health variables: sex, age, place of birth and residence, job, level of studies, level of income and usual residence, weight, height, self-perceived degree of health, the presence of diseases that could modify the diet and the presence of symptoms of eating disorders. Additionally, it collects data related to nutritional habits and frequency of consumption of different foods, sedentary lifestyles, and the practice of physical activity. Finally, it focuses on health-related social habits such as sleep, smoking, and alcohol consumption.

Moreover, most of the variables were qualitative, with the possibility of choosing one of the multiple options: Sex (male or female), place of birth and residence, level of studies (all possible levels of studies in the Spanish education system), level of income (different salary steps) and all frequencies of food and drink consumption where one could choose between increasing frequency steps. The same was also valid for sedentary lifestyle, hours of sleep, and tobacco consumption, where one could choose between different categories. Nevertheless, to work with them quantitatively, the food habits and lifestyle variables were categorized on a Likert scale from 1 to 4 points, following the criteria indicated in Table 1.

**Table 1:** Categorization of beverage consumption variables and the health and lifestyle variables.

Variable	Category	Score
Soft drinks	Never and very rarely (2 glasses max. Per month)	1
	One glass per week and 2 or more glasses per week	2
	2 glasses or less every day	3
	3 to 5 glasses and more than 5 glasses every day	4
Smoking	Non-smoker	1
	Light smoker (less than 5 cigarettes per day)	2
	Moderate smoker (6–15 cigarettes per day)	3
	Severe smoker (more than 16 cigarettes per day)	4
Sedentary lifestyle	Less than 7 h	1
	Between 7 and 9 h	2
	Between 9 and 11 h	3
	More than 11 h	4

Other variables had a continuous quantitative value, such as age, weight, height, and minutes of exercise per week, and others had a discrete quantitative value in the Likert scale format, such as self-reported level of health.

The results of the food frequency variables were used to calculate the IASE (Healthy Eating Index for the Spanish population), which was calculated using a reduced version of the index validated by Norte and Ortiz (33). The index assigns a score of 10 to behavior that meets the recommendations proposed by the Spanish Society of Community Nutrition (34) (SENC). The maximum score of the index is 73. IASE distinguishes the degree of healthiness of eating habits in three categories: ‘Healthy’ to those values of the nutritional index between 58.4 and 73, the classification of ‘Needs changes’ to those between 36.5 and 58.4, and ‘Unhealthy’ to those below 36.5. Table 2 shows the categorization of variables used for the IASE.

**Table 2:** Criteria used for the calculation of the IASE.

Conversion table applied for IASE					
Variables	Score				
	10	7.5	5	2.5	0
Fruit	1 piece/portion per day, 2 to 4 portions per day, 5 or more pieces per day				Never or rarely
Vegetables	Every day	5 or more pieces per week and Between 2 and 4 pieces per week	1 piece/ration per week		Never or rarely
Cereals	Every day	3 or more times a week	1–2 times a week		Never or rarely
Milk	Every day	3 or more times a week	1–2 times a week		Never or rarely
Medium between white and red meat	1–2 times a week	3 or more times a week		Every day	Never or very rarely
Legumes	1–2 times a week	3 or more times a week		Every day	Never or very rarely
Soft drinks	Never or rarely	Very few times (2 times maximum per month)	One glass per week	2 or more glasses per week	Two glasses or less every day and Between 3 and 5 glasses every day and More than 5 glasses every day
Variety	2 points if each of the daily recommendations is met, 1 point if each of the weekly recommendations is met.				

### 2.4 Data analysis

Concerning data preparation, the data from the questionnaires were collected and stored in a database designed specifically for this study, and then, it proceeded to check if there were any erroneous data as a result of data entry or if there were outliers. Thus, once the information was cleaned, the corresponding descriptive and inferential statistical analysis was carried out. From the final dataset, nine variables were selected because of their interest, such as Sex, Studies, BMI, IASE, Self-perceived health, Soft drinks, Sedentary lifestyle, Sport, and Smoking. Then, individuals with BMI < 14 and BMI > 40 were removed, as they were considered extreme values. Below, discrete variables are shown as absolute values and percentages, and continuous variables are shown as the mean and the standard deviation.

Furthermore, to determine the power of the study, this was carried through the G*Power program (35), performing a post-hoc analysis, with a significance level set at 0.001, and obtaining a maximum power, as the sample size is big enough.

To explore the normality of the data, the Lilliefors Test (Kolmogorov–Smirnov) was used with a significance level of 95%, and the test showed that data do not follow a normal distribution. Thus, the Kruskal-Wallis test (non-parametric version of ANOVA) was performed to compare whether there are differences between the 7 groups for the variable “Studies.”

Computing the effect size for the Kruskal-Wallis test as the eta squared based on the H-statistic allows us to obtain the percentage of variance in the numerical variables explained by Studies. The interpretation values commonly in published literature are 0.01–0.06 (small effect), 0.06–0.14 (moderate effect), and > = 0.14 (large effect) (36).

Notwithstanding, clustering techniques were studied to look for individuals segmented in groups within data. Clustering, in the context of data analysis, is a technique used to group similar objects or records into sets to discover patterns and underlying structures in the data. The goal is to have objects within the same group be more like each other than those in other groups. These groups, known as “clusters,” help simplify and comprehend the data. Thanks to the clustering technique, it is feasible to observe individuals from our sample “naturally” group in which manner and what the variables that discriminate better among them are (37, 38).

When dealing with mixed data that includes variables of different types (quantitative or qualitative) (39), it is necessary to approach clustering in a specific manner. Thus, the optimal is to use the Gower distance, a metric that can handle mixed data by calculating distances between objects while considering the unique characteristics of each feature type.

On the other hand, the K-medoid method, which is outlier-resistant and can reduce the effect of outliers and noise in the data, was also considered. Noise in the data refers to any unwanted or irrelevant information or variability present within a dataset that does not represent meaningful patterns or essential information. This could include outliers (data that significantly differ from the rest of the observations in a dataset), errors, or random fluctuations that might obscure or distort the actual trends or relationships in the data.

The Silhouette Coefficient was calculated as a metric to select the number of components, discerning the advised number of clusters (40). The Silhouette Coefficient is a measure used to evaluate the quality of clustering. It quantifies how well-defined the clusters are in a dataset. It ranges from −1 to 1, where a higher value indicates that the clusters are well-separated. Meanwhile, a lower value suggests that the clusters may overlap or that data points have been assigned to incorrect clusters. The Silhouette Coefficient considers both the cohesion of individuals within clusters and the separation between clusters.

**Figure 1:**
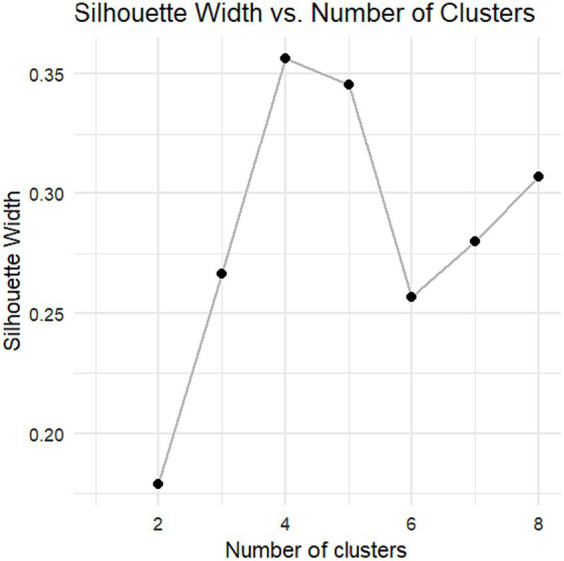
Graphical representation of the Silhouette coefficient according to the number of clusters.

Then, to maximize the Silhouette Coefficient, 4 clusters were selected, with a 0.36 average Silhouette width, as seen in the plot in Figure 1. All these methods were performed with RStudio 4.3.0 (39).

## 3 Results

The final sample consisted of 9,681 valid surveys. Table 3 shows the sociodemographic characteristics of the sample. As observed, 81.05% of the respondents are female, while 18.95% are male. The majority of the sample (38.16%) has a degree, followed by 24.59% who have a master’s degree, 20.86% have a secondary education, 13.65% have a vocational education, only 0.14% (14 people) have no education, and 1.58% have only primary education, while 1.02% of young people have a doctorate degree. The BMI prevalence according to WHO (41) categories in our database is 6.88% Underweight, 70.72% Normal weight, 16.25% Pre-obesity, 4.67% Obesity class I, and 1.48% Obesity class II.

**Table 3:** Sociodemographic characteristics of the sample.

	Mean (SD) or n (%)
Male	1835 (18.95%)
Female	7,846 (81.05%)
Age (years)	24.35 (3.36)
Male Age (years)	24.28 (3.40)
Female Age (years)	24.37 (3.50)
Level of education	Total
No studies	14 (0.14%)
Primary	153 (1.58%)
Secondary	2019 (20.86%)
Professional training	1,321 (13.65%)
Degree	3,694 (38.16%)
Master	2,381 (24.59%)
PhD	99 (1.02%)

Table 4 compares the behavior of nutrition and lifestyle variables concerning sex. For all variables, there are significant differences between the sexes. Men have a higher BMI than women and a higher IASE; they also have a higher level of self-perceived health, spend more hours sitting, and consume sugar-sweetened beverages more frequently. On the other hand, they smoke less and do more sport.

**Table 4:** Comparison of nutrition and health habits differentiated by gender.

	Mean (SD)		
	Male	Female	*p*-value
BMI	24.08 (3.74)	22.81 (4.30)	<0.001
IASE	53.98 (9.89)	53.31 (9.76)	<0.001
Self-perceived health	4.07 (0.76)	3.78 (0.82)	<0.001
Sedentary lifestyle	2.37 (0.88)	2.34 (0.86)	0.036
Sport (min)	288.47 (230.15)	147.31 (167.17)	<0.001
Smoking	2.26 (0.81)	2.32 (0.76)	0.027
Soft drinks	1.68 (0.16)	1.65 (0.70)	<0.001

Table 5 represents the mean values and standard deviation of the health and habit variables concerning the different categories of the educational level of the respondents. BMI and IASE (Healthy Eating Index for the Spanish population) might be the most striking values. It can be observed how the mean Body Mass Index varies by almost 3 points between the subjects with no education (BMI = 25.14) and those with higher education, university, or master’s degree (BMI = 22.74).

**Table 5:** Comparison of nutrition and health habits regarding the level of education.

Mean (SD)							
	No studies	Primary	Secondary	Prof. training	Degree	Master	PhD
BMI	25.14 (5.96)	24.08 (4.91)	22.45 (3.66)	24.31 (4.66)	22.74 (3.68)	22.74 (3.62)	23.22 (3.48)
IASE	46.89 (13.43)	46.23 (11.61)	53.44 (9.38)	50.40 (10.58)	53.85 (9.49)	54.52 (9.12)	55.44 (8.46)
Self-perceived health	3.93 (1.00)	3.48 (0.85)	3.84 (0.80)	3.64 (0.88)	3.85 (0.80)	3.94 (0.77)	4.16 (0.67)
Sedentary lifestyle	1.36 (0.63)	1.46 (0.83)	1.75 (0.88)	1.49 (0.81)	1.64 (0.87)	1.69 (0.88)	1.88 (0.82)
Sport (min)	266.79 (196.61)	151.27 (203.02)	175.22 (199.80)	175.12 (207.72)	169.70 (182.58)	181.91 (177.51)	193.18 (172.80)
Smoking	2 (1.11)	1.67 (0.97)	1.19 (0.53)	1.37 (0.75)	1.19 (0.55)	1.16 (0.51)	1.09 (0.35)
Soft drinks	1.79 (1.05)	1.78 (0.92)	1.43 (0.65)	1.62 (0.81)	1.39 (0.63)	1.34 (0.61)	1.17 (0.50)

The variations in IASE are also important, going from a minimum value of 46.23 for subjects with primary education to a maximum value of 55.44 for subjects with a doctorate. Although both values are in the IASE range where it is necessary to make changes in nutrition [36.5–58.4], this is a variation of more than 9 points on a scale where the maximum is 73. It is also possible to observe that people with the highest level of education are very close to the range of healthy nutrition habits.

To test for statistically significant differences between the values of the health and habits variables and the different levels of study, the Kruskal-Wallis test and the Wilcoxon test with Hochberg adjustment were performed to study in more detail among which groups the differences were found. For all variables, a significant *p*-value (*p*-value <0.001) was encountered, indicating therefore the existence of significant differences in at least two different levels of education for all health and habit variables explored. For a more detailed view, the pairwise comparisons have been calculated in Appendix A.

**Figure 2:**
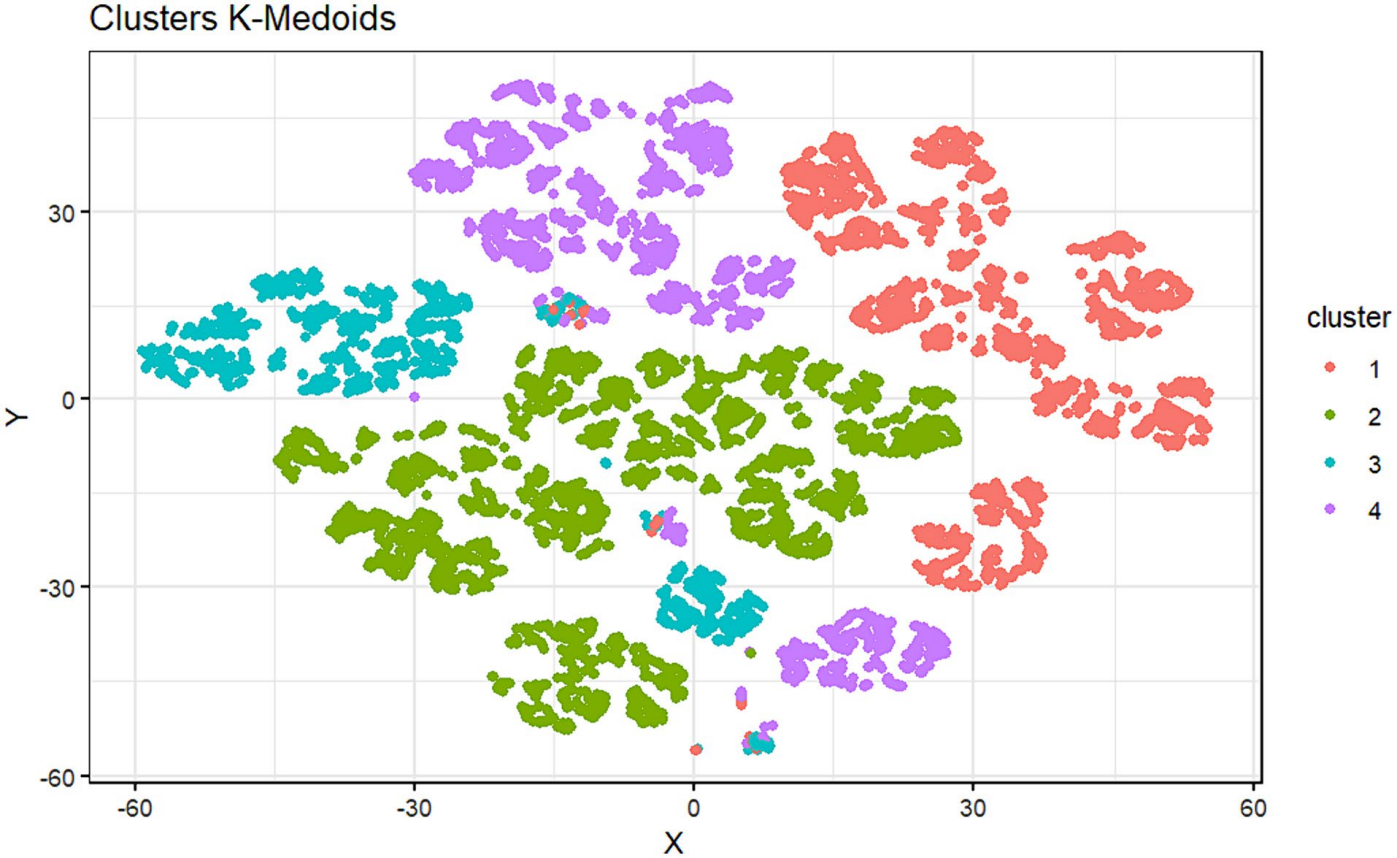
Graphical representation of the distribution of subjects in each cluster.

For the clustering analysis, 4 clusters were chosen. The Silhouette Coefficient per cluster is 0.38 (Cluster 1), 0.39 (Cluster 2), 0.27 (Cluster 3), and 0.32 (Cluster 4). At first sight, it is impossible to appreciate a considerable misclassification of individuals. On the contrary, it is possible to observe that cluster 2 is the best-defined one (as it has the highest Silhouette Coefficient). On the contrary, cluster 3 is the worst-defined cluster, with more misclassified individuals. Figure 2 shows the distribution of the four resulting clusters, and it can be seen how the data have been grouped in a well-defined way in 4 different groups or patterns.

Table 6 describes the structure of each cluster, indicating the mean and median obtained for each nutrition and habits variable explored and specifying the subjects who have fallen into each cluster according to their level of education.

**Table 6:** Median and mean values of the variables in each cluster.

	**CLUSTER 1** **N=2430** No studies = 5 Primary = 24 Secondary = 0 Professional training= 0 Degree = 0 Master = 2381 PhD = 20		**CLUSTER 2** **N=3706** No studies = 0 Primary = 9 Secondary = 0 Professional training=0 Degree = 3694 Master = 0 PhD = 3		**CLUSTER 3** **N=1419** No studies = 5 Primary = 78 Secondary = 0 Professional training = 1321 Degree = 0 Master = 0 PhD = 15		**CLUSTER 4** **N=2126** No studies = 4 Primary = 42 Secondary = 2019 Professional training=0 Degree = 0 Master = 0 PhD = 61	
	**Median**	**Mean**	**Median**	**Mean**	**Median**	**Mean**	**Median**	**Mean**
**Body Mass Index (BMI)**	22.22	22.86	22.04	22.73	23.18	24.26	21.88	22.55
**Nutritional Index (IASE)**	56.00	54.66	55.00	53.85	51.25	49.81	55.00	53.32
**Self-perceived health**	4	3.95	4	3.85	4	3.64	4	3.84
**Sedentary lifestyle**	1	1.68	1	1.65	1	1.46	2	1.79
**Sport (min)**	135	183.5	135	169.5	90	172.8	135	174.9
**Soft drinks**	1	1.34	1	1.39	1	1.64	1	1.43
**Smoking**	1	1.16	1	1.19	1	1.39	1	1.19

Cluster 1 comprises 2,430 subjects, most of whom have completed a master’s degree. No individuals have a bachelor’s degree, secondary education, or vocational training. Cluster 2 groups 3,706 subjects, and it is wholly constituted of people who studied for a bachelor’s degree. Cluster 3 (*n* = 1,419) is composed of people who studied professional training. Finally, Cluster 4 contains 2,126 subjects and is comprised of young people with secondary studies and no professional training, bachelor’s degree, or master’s degree.

## 4 Discussion

Firstly, Table 4 shows the comparison between the sexes. Men have a higher average BMI (24.08) than women (22.81), although in both groups, the average BMI is in the normal weight range. This data is in line with previous studies in the literature (42, 43) and, given that it is observed that men dedicate almost twice as much time to physical activity as women (288 vs. 147 min a week), it could be due to a higher percentage of muscle mass in men compared to women.

From this result, it can be inferred that young women are very close to (or do in the case of high-intensity sport) the physical activity recommended by the WHO (41): 150 min per week of moderate-intensity sport or 75 min of high-intensity sport. Young men, on the other hand, far exceed this threshold. Given the proven wide-ranging benefits of physical activity (44–46), it is excellent news in terms of health to see young men devoting ample time to physical activity.

The higher frequency of physical activity in men than in women may partly explain men’s higher consumption of sugar-sweetened beverages. The practice of physical activity leads to increased sweating with a consequent need to rehydrate the body and replenish lost fluids and minerals (47, 48). Although it may not be the healthiest way, many young men consume sugary drinks after sport.

Table 5 shows the means comparison of the variables between the different levels of education. As can be seen from the values obtained in such comparisons (Appendix A), people with secondary education have a significantly different BMI compared to those with higher education, and the same is observed for people with vocational training. A similar trend is found for the IASE value, which is significantly higher for people with doctoral, master’s, and bachelor’s degrees than those with less education. Also, sedentary behavior is higher for people with higher education than those with more basic education. Although it will be discussed later, this seems to indicate that the level of education influences the health habits of the population.

Table 6 shows the grouping into clusters, from which the groups are relatively homogeneous regarding the educational level of the subjects in each cluster. This again seems to indicate that the level of education of the individuals has some influence on their health and lifestyle habits, contributing to the fact that, in general, the health clusters are different.

The clustering technique has allowed us to find whether there are different patterns in the data that can group individuals according to a series of characteristics. As can be seen, the cluster generated from the variables of interest is made up of four different groups for their health patterns. Furthermore, the most curious thing is that these four groups practically correspond to four different levels of education. Although, there are indeed individuals who, despite having one level of education, have a different health pattern.

Curiously, the classes generated from health-related variables coincide closely with the different levels of education. Therefore, it is possible to see that the level of education seems to have a significant impact on health, and we observe that a higher level of education is directly related to healthier habits.

Furthermore, cluster 3 is the one that includes the subjects with the lowest level of education, given that it has grouped all the subjects with a level of vocational training and most of those with primary education. In this cluster, the BMI is the highest, standing close to the threshold of 25, where a person begins to be considered overweight (49). The healthy nutrition index IASE is also the lowest, differing by 4–5 points from the other clusters, indicating that the nutritional habits of these people are the least healthy. Also, other aspects related to health have more negative results. These subjects consume sugary drinks more frequently. The high consumption of sugar-sweetened beverages has been shown to be detrimental to health (50, 51), and have a higher addition to tobacco.

On the other hand, concerning physical activity, the average time spent by the members of each cluster is similar. The only habit that seems healthier for people with vocational and primary education is a sedentary lifestyle, indicating that these people spend less time sitting on average.

This healthy trend in habits also significantly impacts people’s perception of their state of health; in fact, the level of self-perceived health is significantly higher in people with higher education than in people with primary education.

Such results are in line with the literature showing that educational level has a significant impact on healthy lifestyle choices (52). Education is a unique dimension of social status that influences health in a varied, cumulative, and uniformly positive way. Educational attainment is an important indicator of future socio-economic position and influences other achieved social statuses such as occupation and personal income.

Generally, people with higher education are less likely to abuse alcohol, exercise more, and eat healthier food than the average population (53). Finally, education and health literacy strongly influence healthy behaviors, and less educated individuals have shorter survival and spend more years of life with a disability (54).

Given the importance of health education and healthy lifestyles, this opens an essential field of public health intervention, where actions could be varied.

On the one hand, one could intervene to facilitate access to higher education for more people who may be unable to do so due to lack of financial resources, time, or adequate knowledge. One measure could be for the state and local government to provide more grants and scholarships for low-income people. Another measure would be for universities and other higher education institutions to offer a broader range of class schedules. For example, in the evening or in different modalities, blended or online, to facilitate participation for those who cannot attend lessons in the morning due to work or family responsibilities. Finally, creating leveling courses for those not considering university studies because they need a solid training base for access could be considered.

On the other hand, it would be possible to intervene in the quality of these higher education courses, to highlight not only the technical aspects of each degree but also a series of cross-cutting subjects that directly affect the health and well-being of the population. We are in a society where one of the most serious epidemics is obesity (55, 56) with all its negative consequences (57, 58). It seems essential to introduce cross-cutting subjects or specific courses in the curricula of the different degree courses that impact the training in nutrition and healthy habits of university students. Prevention is the most effective weapon against the growth of this serious public health problem.

Analyzing the overall health habits of young Spaniards allows us to affirm that alcohol is not an issue of concern given that most respondents for all groups consume it sporadically, as is tobacco consumption, where most young people do not smoke or smoke only occasionally. Physical activity is also above the recommended 150 min per week to obtain positive health benefits (41). These healthy habits are reflected in the BMI, within the normal weight category, and in the self-perceived health status which is in the *‘Good’* to *‘Very good’* range for all groups analyzed.

Instead, changes in sedentary lifestyles are necessary, as all groups spend between 7 and 9 h sitting, which is more than the 6 h a day considered healthy. Finally, regarding nutrition, it is found that all groups need to change their food consumption habits. Using the same criteria as in the Norte and Ortiz study to classify the IASE (33), it is observed that the mean IASE values ranged from a maximum of 55.44 to a minimum of 46.89, both of which were in the range suggesting dietary changes.

This result shows where training efforts should be more specifically focused: in the field of nutrition. In addition to the theoretical training that could be provided in the curricula, as indicated above, several complementary initiatives could be implemented. This comprehends courses on quick and easy cooking for students and young people, who too often abuse ready-made meals and ultra-processed foods (59, 60). Moreover, it might be interesting to promote greater legislative control over additives, preservatives, and 4th and 5th-range products used in the food industry. Finally, it should be crucial to demand greater clarity and transparency in the labeling of products and the training of consumers. Therefore, they could know how to interpret labels so that they are always aware of what they are buying and ingesting, among other things.

### 4.1 Study strengths and limitations

The main strength of this study lies in the size of the sample, which includes a broad representation of young people throughout the Spanish population. One of the weaknesses of the study lies in the type of sampling used: a self-administered questionnaire disseminated in snowball networks. This type of sampling is very powerful because it makes it possible to reach a substantial population. Nevertheless, due to the fact of not answering in front of the interviewer, it is not possible to ensure the identity of the respondent and, sometimes, that the respondent has understood the question correctly. This possible bias has been considered in the design of the questionnaire to minimize it by formulating straightforward questions and answers.

Another bias that should be highlighted is gender, with 81.05% of respondents being female. It is a trend also found in other studies that women are more likely to participate in surveys, mainly if they are focused on health and well-being. To partially alleviate this trend, a conscious effort was made to recruit male representatives for the study, and in the end, 1,835 responses from men were obtained, representing a more than significant sample of this genre.

### 4.2 Areas for further research

We believe that this study could be the starting point for future research in this field and that it might be interesting to investigate the impact of education and habits on children and adolescents rather than on young adults as has been done. It might also be interesting to disseminate another questionnaire focusing on a male sample.

Another future study that could certainly be of interest could be the design and implementation of specific training programs on nutrition and habits in the young population to measure the impact of this training on health improvement.

It could also be interesting to extend this study to other countries, analyzing whether the conclusions obtained for Spanish young people can be extrapolated to other regions and showing the differences. In this respect, the authors are in the process of translating and culturally adapting the instrument to Italian and Chilean. They will soon be able to collect data in those countries as well.

## 5 Conclusion

The main results of this work indicate that, in general, people with a higher level of education have healthier lifestyles and better health. It shows how crucial it is for young people to have access to higher levels of education and good training to acquire healthy habits and a healthy lifestyle.

Finally, we found that, in general, the health habits of the young Spanish population seem pretty healthy. However, there is a need for improvement in those aspects related to nutrition and food, indicating a clear field for action on public health interventions.

## Data availability statement

The raw data supporting the conclusions of this article will be made available by the authors, without undue reservation.

## Ethics statement

The studies involving humans were approved by Ethics Research Committee of the Catholic University of Valencia (approval code UCV/2019–2020/152). The studies were conducted in accordance with the local legislation and institutional requirements. The participants provided their written informed consent to participate in this study. Written informed consent was obtained from the individual(s) for the publication of any potentially identifiable images or data included in this article.

## Author contributions

ES: Conceptualization, Formal analysis, Investigation, Methodology, Project administration, Resources, Supervision, Validation, Writing – original draft, Writing – review & editing. JP: Formal analysis, Investigation, Methodology, Supervision, Validation, Writing – original draft, Writing – review & editing. EL: Conceptualization, Data curation, Investigation, Methodology, Software, Validation, Visualization, Writing – original draft, Writing – review & editing. GO: Conceptualization, Methodology, Supervision, Validation, Writing – review & editing. AF: Formal analysis, Funding acquisition, Methodology, Supervision, Validation, Writing – review & editing.
