# Supplementary material for: Analysis of the influence of educational level on the nutritional status and lifestyle habits of the young Spanish population
Source: Front Public Health. 2024 Apr 8;12:1341420. doi: 10.3389/fpubh.2024.1341420 (PMC11033505; doi:10.3389/fpubh.2024.1341420)
Supplement: Supplementary file 1 [file Data_Sheet_1.PDF]

Appendix A

Tables [A1-A7] show the results of the pairwise comparisons between the healthy variables and the different levels of education.

Table A1. Pairwise comparisons for BMI with Hochberg P-value adjustment method.

	Secondary	PhD	Primary	Professional Training	Bachelor's Degree	Master's Degree
PhD	0.76					
Primary	**0.002**	0.78				
Professional Training	**<0.001**	0.78	0.78			
Bachelor's Degree	**0.01**	0.72	0.04	**<0.001**		
Master's Degree	**<0.001**	0.78	0.15	**<0.001**	0.78	
No Studies	0.78	0.78	0.78	0.78	0.78	0.78

Table A2. Pairwise comparisons for IASE with Hochberg P-value adjustment method.

	Secondary	PhD	Primary	Professional Training	Bachelor's Degree	Master's Degree
PhD	0.41					
Primary	**<0.001**	**<0.001**				
Professional Training	**<0.001**	**<0.001**	**<0.001**			
Bachelor's Degree	0.41	0.59	**<0.001**	**<0.001**		
Master's Degree	**0.002**	0.79	**<0.001**	**<0.001**	0.16	
No Studies	0.41	0.30	0.79	0.79	0.41	0.33

Table A3. Pairwise comparisons for Self-perceived health with Hochberg P-value adjustment method.

	Secondary	PhD	Primary	Professional Training	Bachelor's Degree	Master's Degree
PhD	**<0.001**					
Primary	**<0.001**	**<0.001**				
Professional Training	**<0.001**	**<0.001**	0.14			
Bachelor's Degree	0.77	**<0.001**	**<0.001**	**<0.001**		
Master's Degree	**<0.001**	**0.04**	**<0.001**	**<0.001**	**<0.001**	
No Studies	0.77	0.77	0.33	0.77	0.77	0.77

Table A4. Pairwise comparisons for Soft drinks with Hochberg P-value adjustment method.

	Secondary	PhD	Primary	Professional Training	Bachelor's Degree	Master's Degree
PhD	**<0.001**					
Primary	**<0.001**	**<0.001**				
Professional Training	**<0.001**	**<0.001**	0.33			
Bachelor's Degree	0.14	**<0.001**	**<0.001**	**<0.001**		
Master's Degree	**<0.001**	**0.04**	**<0.001**	**<0.001**	**0.002**	
No Studies	0.76	**0.02**	0.85	0.85	0.69	0.42

Table A5. Pairwise comparisons for Sedentary lifestyle with Hochberg P-value adjustment method.

	Secondary	PhD	Primary	Professional Training	Bachelor's Degree	Master's Degree
PhD	0.36					
Primary	**<0.001**	**<0.001**				
Professional Training	**<0.001**	**<0.001**	0.92			
Bachelor's Degree	<0.001	0.006	**0.02**	**<0.001**		
Master's Degree	**0.10**	0.07	**0.0015**	**<0.001**	**0.07**	
No Studies	0.53	0.02	0.92	0.92	0.92	0.80

Table A6. Pairwise comparisons for Sport with Hochberg P-value adjustment method.

	Secondary	PhD	Primary	Professional Training	Bachelor's Degree	Master's Degree
PhD	0.49					
Primary	0.31	**0.03**				
Professional Training	0.66	0.27	0.56			
Bachelor's Degree	0.66	0.49	0.21	0.65		

Master's Degree	**0.003**	0.66	**0.004**	**<0.001**	**0.001**	
No Studies	0.49	0.66	0.33	0.49	0.49	0.58

Table A7. Pairwise comparisons for Smoking with Hochberg P-value adjustment method.

	Secondary	PhD	Primary	Professional Training	Bachelor's Degree	Master's Degree
PhD	0.43					
Primary	**<0.001**	**<0.001**				
Professional Training	**<0.001**	**<0.001**	**<0.001**			
Bachelor's Degree	0.96	0.43	**<0.001**	**<0.001**		
Master's Degree	0.43	0.51	**<0.001**	**<0.001**	0.38	
No Studies	**<0.001**	**<0.001**	0.51	0.05	**<0.001**	**<0.001**
